# Comparative analysis of methodologies for predicting overall survival in patients with non‐small cell lung cancer based on the number and rate of resected positive lymph nodes: A study based on the SEER database for 2010 through 2019

**DOI:** 10.1111/crj.13699

**Published:** 2023-09-18

**Authors:** Qiang Guo, Sheng Hu, Silin Wang, Lang Su, Wenxiong Zhang, Jianjun Xu, Yiping Wei

**Affiliations:** ^1^ Department of Thoracic Surgery The Second Affiliated Hospital of Nanchang University Nanchang Jiangxi China

**Keywords:** comparative study, NSCLC, number of positive LNs, positive LN rate, SEER

## Abstract

**Background:**

Lymph node (LN) metastasis is crucial in non‐small cell lung cancer (NSCLC) prognosis and treatment, but the TNM system lacks LN quantity consideration. Our goal is to investigate the role of positive LNs (nPLN) and positive LN rate (LNR) in overall survival (OS) and assess whether they offer higher value in prognostic assessment of NSCLC than N‐stage.

**Methods:**

Patients were stratified into four subgroups using X‐Tile software. Statistical analysis was conducted using the Kaplan–Meier method, univariate analysis, and multivariate Cox regression analysis. Model performance was evaluated using the Harrell consistency index (C‐index), Akaike information criterion (AIC), and Bayesian information criterion (BIC). The prognostic performance of the nodal classification was validated using overall survival as the endpoint.

**Results:**

The survival curves demonstrate distinct disparities between each nPLN and LNR category. A pronounced trend toward deteriorating overall survival from N‐PLN 1 to N‐PLN 2+ was observed across all tumor size categories. However, the differences between each LNR category were only significant for tumors ≤3 cm and 5–7 cm. Notably, both nPLN and LNR classifications displayed a higher C‐index, lower AIC, and lower BIC compared with the N staging. Furthermore, the LNR classification provided superior prognostic stratification when compared with the nPLN classification.

**Conclusions:**

Our results demonstrate that nPLN and LNR classifications may offer improved prognostic performance compared with the current N classification for LN‐positive NSCLC patients. Nonetheless, more studies are needed to assess the feasibility of incorporating these classifications into the next TNM staging system.

## Graphical abstract

In this paper, we report on that the significant prognostic value of number of positive lymph nodes (nPLN) and lymph nodes rate (LNR) in predicting survival outcomes for LN‐positive NSCLC patients who undergo surgery and these classifications may offer improved prognostic performance compared with the current N classification.

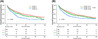

## 1 INTRODUCTION

The presence and location of lymph nodes (LNs) metastasis hold considerable influence on prognosis and treatment selection in non‐small cell lung cancer (NSCLC) patients. The status of LNs in many malignant tumors depends on the anatomical location and the number of involved nodes. However, the TNM system's classification of LNs status in NSCLC solely considers the anatomical location involved and not the number of LNs.^1^, ^2^, ^3^, ^4^, ^5^, ^6^ The collection of LNs during pulmonary hilum and mediastinum surgeries and their comprehensive examination by pathologists determine the number of LNs and stations for staging. Nevertheless, in practice, surgeons subjectively assign names to LN stations, leading to possible errors in N staging. Additionally, cases of LN sampling instead of dissection by surgeons may also affect staging. Alternative LN evaluation methods such as the number of positive LNs (nPLNs) and the rate of positive LNs (LNR, the ratio of nPLNs to the number of LNs resected) may provide more reliable prognostic outcomes for LN‐positive NSCLC patients.^7^, ^8^ Lung cancer, unlike other solid tumors, is categorized entirely by the location of LNs.^9^ However, the nPLN has been incorporated into TNM staging for breast, gastric, and colorectal cancer.^1^, ^10^, ^11^, ^12^ In recent years, numerous studies have explored nPLN and LNR as reliable prognostic factors for NSCLC beyond the limitations of TNM staging.^3^, ^13^ These studies have analyzed the number of resected LNs or the ratio of metastatic LNs to resected LNs, yielding encouraging results for NSCLC and other cancers. However, despite these promising findings, the eighth edition of TNM classification for NSCLC has not altered the methodology used to determine N‐stage.^1^, ^14^ Moreover, the value of nPLN, LNR, and current N classification in predicting the prognosis of NSCLC patients has not yet been verified in population‐based studies. To address these issues, we have undertaken a study utilizing a large multicenter database of 1410 patients. Our goal is to investigate the role of nPLN and LNR in overall survival (OS) and assess whether they offer higher value in prognostic assessment of NSCLC than N‐stage. Ultimately, our aim is to incorporate these factors into the TNM staging system.

Over the past decade, the global thoracic oncology community has been fortunate to benefit from the highly reliable clinical practice guidelines for lung cancer staging provided by the American Thoracic Society and the European Society of Thoracic Surgeons (ESTS).^15^, ^16^, ^17^, ^18^ These guidelines are the result of extensive research and a large body of literature and have established a standard approach to lung cancer staging in most regions of the world. Emphasizing the importance of achieving the highest level of certainty in clinical and pathological staging, the guidelines recommend a careful combination of imaging, endoscopic examinations, minimally invasive surgery, and thorough intraoperative staging, as well as sound pathological examination of tissue biopsies, fluids, and resection specimens. Ideally, these examinations should be performed in sequence and with increasing invasiveness. Staging at diagnosis serves as the foundation for planning initial treatment, while staging after tumor resection provides the strongest prognostic indicator and the necessary information for determining postoperative treatment. The International Union Against Cancer (UICC), the American Joint Committee on Cancer, and the International Association for the Study of Lung Cancer (IASLC) collaborated to release the eighth edition of the Tumor, Node, and Metastasis (TNM) classification.^15^, ^19^

Recent research has revealed that metastases can occur at N2 stations without N1 involvement. Furthermore, individuals who bypass N2 stage illness typically exhibit better prognoses than those who do not.^20^, ^21^, ^22^ This highlights that the total tumor burden may serve as a more precise prognostic indicator than the physical location of LN involvement. However, the current LN staging system fails to fully account for the tumor burden of regional LNs. This is because small metastatic LNs, including micrometastases, are frequently grouped into the same category as grossly involved multiple LNs. Consequently, individuals with the same N status, particularly those with N1 or N2 disease, may have varying prognoses. To address this issue, it is critical to identify alternative N staging approaches that provide more precise prognostic stratification. Recent research has identified two promising alternative measures: nPLN and LNR.^23^, ^24^, ^25^, ^26^, ^27^ It has been demonstrated as a powerful independent prognostic indicator in several solid tumors, including breast, colorectal, and head and neck cancers.^11^, ^12^, ^24^, ^28^, ^29^ On the other hand, LNR is the ratio of the number of positive LNs to the total number of LNs examined. This measure has also shown to be a strong predictor of survival in various tumor types, such as gastric, pancreatic, and esophageal cancers. While LN involvement remains a crucial factor in cancer staging, it is apparent that the current system has limitations in accurately reflecting tumor burden and predicting prognosis. Alternative measures such as nLNR and LNR hold great promise in enhancing prognostic stratification and guiding treatment decisions for patients with solid tumors.

## 2 MATERIALS AND METHODS

### 2.1 Data source

**FIGURE 1:**
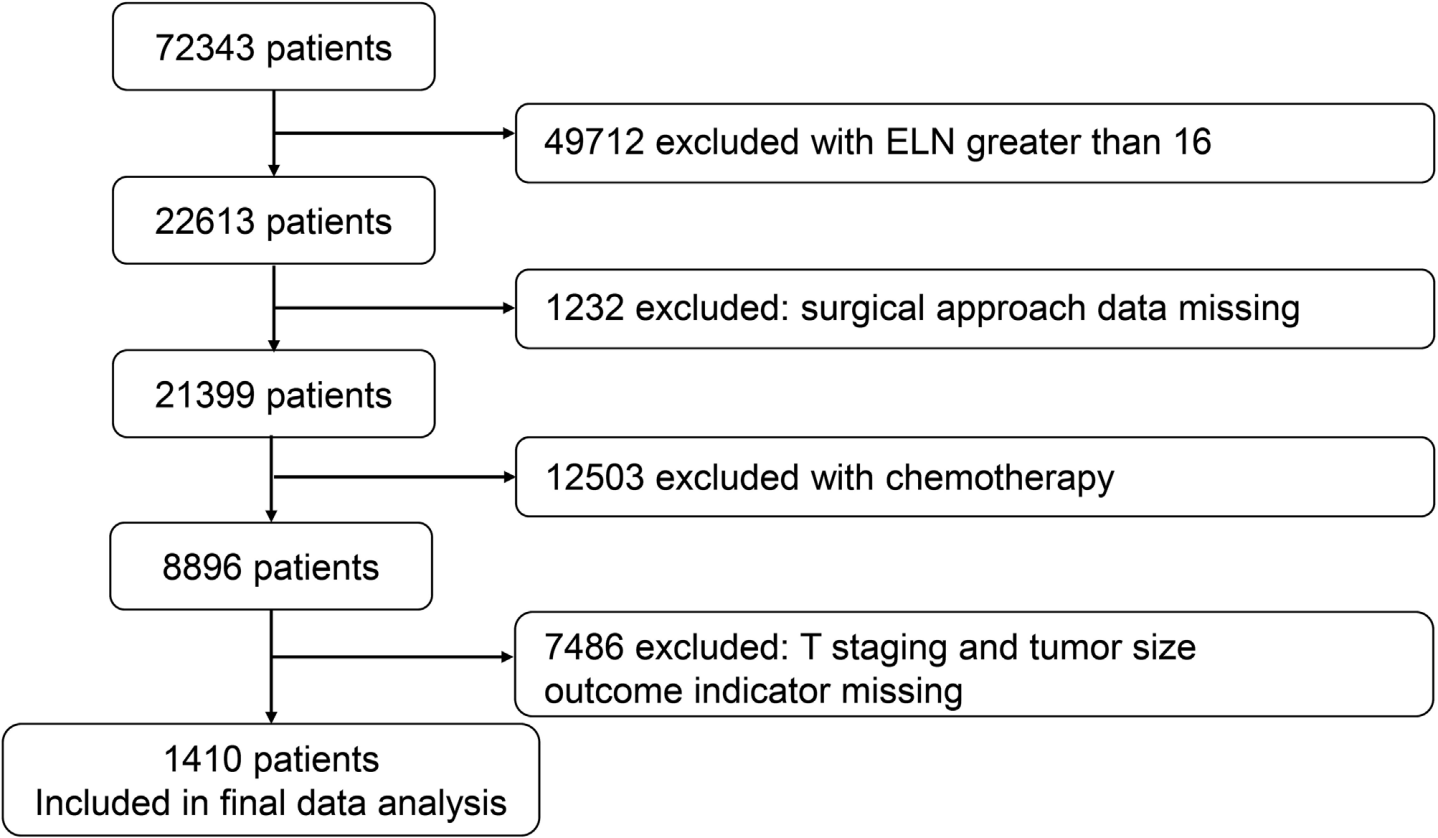
Flow chart. ELN: examined lymph nodes.

We extracted data from the Surveillance, Epidemiology, and End Results Program (SEER) 17 Registry study database, which is the most recent and contains data from 2010 to 2019. The SEER 17 database covers SEER 17 registry centers across the United States, including major cities and rural areas. Cancer data collection begins by identifying people with cancer who have been diagnosed or received cancer care in hospitals, outpatient clinics, radiology departments, doctors' offices, laboratories, surgical centers, or from other providers (such as pharmacists) who diagnose or treat cancer patients. All 50 states have laws requiring newly diagnosed cancers to be reported to a central registry. Cancer registries review these reported cases and determine whether such information is reportable according to law. If so, registries pull cancer information as required by the North American Association of Central Cancer Registries' (NAACCR) Data StandardsExternal Web Site Policy from the medical records for those cases. Our study included patients with stage N1 or N2 NSCLC who had undergone lobectomy or total pneumonectomy. We excluded patients with multiple primary cancers, missing number of examined LNs (ELN) counts, clinical features, and information on positive LNs, T‐stage, and tumor size. Patients who received chemotherapy were also excluded to ensure the inclusion of patients who did not undergo neoadjuvant therapy. We only analyzed patients who underwent surgery and had a minimum of 16 LNs removed, as recommended in previous studies. Figure 1 provides a detailed explanation of our inclusion and exclusion criteria. Overall, our analysis included a large multicenter database of 1410 patients, providing a robust dataset for investigating the role of nPLN and LNR in prognostic assessment of NSCLC. The study protocol was approved by the ethics committee of the Second Hospital of Nanchang University in China, ensuring that the study was conducted in accordance with ethical principles and regulations.

### 2.2 Patients and outcomes

The case list was generated using SEERStat version 8.4.0, a software tool available at SEER.cancer.gov/seerstat. The dataset comprised variables including gender, age, histological type, grade, stage, T‐stage, N‐stage, type of surgery, and postoperative radiotherapy. Histological types were classified as squamous cell carcinoma (SCC), adenocarcinoma (ADC), and other. Surgical procedures were recorded as lobectomy or pneumonectomy. To assess the impact of nPLN and LNR on the prognosis, N1 and N2 patients were divided into two subgroups based on their respective nPLN or LNR values. This resulted in the stratification of all patients into four subgroups. Using X‐Tile software (Figure S1), the optimal cut‐off values for nPLN and LNR were determined. We categorized our patient cohort into four groups based on optimal cutoff values for nPLN and LNR: N1‐PLN 1 (with one metastatic LN), N1‐PLN 1+ (with more than one metastatic LN), N2‐PLN 1–2 (with 1–2 metastatic LNs), and N2‐PLN 2+ (with more than 2 metastatic LNs). Similarly, we defined four groups based on LNR values: R1‐LNR ≤0.17 (LNR less than or equal to 0.17), R1‐LNR >0.17 (LNR greater than 0.17), R2‐LNR ≤0.38 (LNR less than or equal to 0.38 for), and R2‐LNR >0.38 (LNR greater than 0.38 for). The primary outcome of the study was OS. The SEER statistical procedure calculated survival time in months, and the study cut‐off date was December 31, 2020. It is important to note that the number of days in a month was determined to be 365.24/12.

### 2.3 Statistical analysis

We conducted an investigation into the association between OS and various node classifications using the Kaplan–Meier (KM) method, followed by a comparison of survival rates between groups using the log‐rank test. In order to enhance the accuracy of survival prediction, we developed a novel approach to node classification, which involves grouping categories with similar survival curves into a single category (nPLN and LNR classification). We used univariate analysis to explore the relationship between each covariate and OS and entered each node classification into separate multivariate Cox regression analyses, ensuring the proportional risk hypothesis was met by examining log–log plots and Schoenfeld residual tests. We evaluated model performance using the Harrell consistency index, Akaike information criterion, and Bayesian information criterion. Furthermore, we validated the impact of our nodal classification on survival across various tumor size categories and key subgroups, based on the eighth edition TNM staging system. The prognostic performance of our nodal classification was validated using overall survival as the endpoint. We considered statistical significance to be indicated by *P* < 0.05 for all tests.

## 3 RESULTS

### 3.1 Baseline characteristics of study participants, stratified by number of LNs

Our study included a total of 1410 patients with NSCLC, of which 1234 (87.52%) had stage N1 and 176 (12.48%) had stage N2. The baseline characteristics of these patients are summarized in Table 1. Among the patients, 57.02% had N1‐PLN 1, 42.98% had N1‐PLN 1+, 79.50% had N2‐PLN 1–2, and 20.50% had N2‐PLN 2+.

**TABLE 1:** Baseline characteristics of patients with N1 or N2 NSCLC according to N‐PLN.

Characteristics	Total (%)	N1 (%)	N1‐PLN 1 (%)	N1‐PLN 1+ (%)	N2 (%)	N2‐PLN 1–2 (%)	N2‐PLN 2+ (%)
Total	1410 (100%)	1234 (87.52%)	804 (57.02%)	606 (42.98%)	176 (12.48%)	1121 (79.50%)	289 (20.50%)
Age							
<65 years	474 (33.62%)	408 (33.06%)	286 (35.57%)	188 (31.02%)	65 (37.36%)	391 (34.88%)	83 (28.72%)
≥65 years	936 (66.38%)	826 (66.94%)	518 (64.43%)	418 (68.98%)	109 (62.64%)	730 (65.12%)	206 (71.28%)
Sex							
Male	710 (50.35%)	634 (51.38%)	395 (49.13%)	315 (51.98%)	75 (43.10%)	560 (49.96%)	150 (51.90%)
Female	700 (49.65%)	600 (48.62%)	409 (50.87%)	291 (48.02%)	99 (56.90%)	561 (50.04%)	139 (48.10%)
Race							
White	1130 (80.14%)	1004 (81.36%)	657 (81.72%)	473 (78.05%)	124 (71.26%)	904 (80.64%)	226 (78.20%)
Black	130 (9.22%)	111 (9.00%)	72 (8.96%)	58 (9.57%)	19 (10.92%)	101 (9.01%)	29 (10.03%)
Other	150 (10.64%)	119 (9.64%)	75 (9.33%)	75 (12.38%)	31 (17.82%)	116 (10.35%)	34 (11.76%)
Year of diagnosis							
2010–2014	707 (50.14%)	707 (57.29%)	420 (52.24%)	287 (47.36%)	174 (100.00%)	590 (52.63%)	117 (40.48%)
2015–2019	703 (49.86%)	527 (42.71%)	384 (47.76%)	319 (52.64%)		531 (47.37%)	172 (59.52%)
Primary_site							
Upper lobe	756 (53.62%)	672 (54.46%)	432 (53.73%)	324 (53.47%)	84 (48.28%)	605 (53.97%)	151 (52.25%)
Middle lobe	93 (6.60%)	86 (6.97%)	63 (7.84%)	30 (4.95%)	7 (4.02%)	81 (7.23%)	12 (4.15%)
Lower lobe	561 (39.79%)	476 (38.57%)	309 (38.43%)	252 (41.58%)	83 (47.70%)	435 (38.80%)	126 (43.60%)
Grade							
I	217 (15.39%)	186 (15.07%)	154 (19.15%)	63 (10.40%)	30 (17.24%)	190 (16.95%)	27 (9.34%)
II	591 (41.91%)	511 (41.41%)	322 (40.05%)	269 (44.39%)	79 (45.40%)	461 (41.12%)	130 (44.98%)
III–IV	602 (42.70%)	537 (43.52%)	328 (40.80%)	274 (45.21%)	65 (37.36%)	470 (41.93%)	132 (45.67%)
Histology							
SCC	365 (25.89%)	342 (27.71%)	207 (25.75%)	158 (26.07%)	23 (13.22%)	303 (27.03%)	62 (21.45%)
ADC	637 (45.18%)	541 (43.84%)	339 (42.16%)	298 (49.17%)	95 (54.60%)	481 (42.91%)	156 (53.98%)
Other	408 (28.94%)	351 (28.44%)	258 (32.09%)	150 (24.75%)	56 (32.18%)	337 (30.06%)	71 (24.57%)
Operation type							
Lobectomy	1289 (91.42%)	1118 (90.60%)	748 (93.03%)	541 (89.27%)	169 (97.13%)	1028 (91.70%)	261 (90.31%)
Pneumonectomy	121 (8.58%)	116 (9.40%)	56 (6.97%)	65 (10.73%)	5 (2.87%)	93 (8.30%)	28 (9.69%)
Tumor size							
≤3 cm	433 (30.71%)	372 (30.15%)	278 (34.58%)	155 (25.58%)	60 (34.48%)	354 (31.58%)	79 (27.34%)
>3 cm, ≤5 cm	580 (41.13%)	496 (40.19%)	314 (39.05%)	266 (43.89%)	84 (48.28%)	457 (40.77%)	123 (42.56%)
>5, ≤7 cm	152 (10.78%)	142 (11.51%)	87 (10.82%)	65 (10.73%)	9 (5.17%)	123 (10.97%)	29 (10.03%)
>7 cm	245 (17.38%)	224 (18.15%)	125 (15.55%)	120 (19.80%)	21 (12.07%)	187 (16.68%)	58 (20.07%)
Postoperative radiation							
Yes	76 (5.39%)	57 (4.62%)	41 (5.10%)	35 (5.78%)	19 (10.92%)	58 (5.17%)	18 (6.23%)
No	1334 (94.61%)	1177 (95.38%)	763 (94.90%)	571 (94.22%)	155 (89.08%)	1063 (94.83%)	271 (93.77%)
ELN, median (Q1–Q3)	9.00 (6.00–12.00)	9.00 (6.00–12.00)	8.00 (5.00–11.00)	10.00 (7.00–13.00)	9.00 (6.00–12.00)	8.00 (5.00–12.00)	10.00 (7.00–14.00)
Positive lymph notes, median (Q1‐Q3)	1.00 (1.00–2.00)	1.00 (1.00–2.00)	1.00 (1.00–1.00)	2.00 (2.00–4.00)	2.00 (1.00–4.00)	1.00 (1.00–2.00)	4.00 (3.00–5.00)
LNR, median (Q1–Q3)	0.19 (0.11–0.33)	0.17 (0.11–0.31)	0.12 (0.09–0.20)	0.30 (0.20–0.50)	0.33 (0.17–0.50)	0.14 (0.10–0.25)	0.42 (0.29–0.60)

Abbreviations: ADC, adenocarcinoma; ELN, examined lymph node; LNR, lymph nodes rate; Q1–Q3, interquartile range; SCC, squamous cell carcinoma.

The distribution of age, race, gender, tumor primary site, histological type, surgical procedure, tumor size, and postoperative radiotherapy was comparable across all groups. However, N1‐PLN 1+ and N2‐PLN 2+ tumors tended to be larger in size and less likely to be well‐differentiated (Grade I).

In our study population, the median number of ELN was 9 (with quartiles ranging from 6 to 12), the median number of positive LNs was 1 (with quartiles ranging from 1 to 2), and the median LNR was 0.19 (with quartiles ranging from 0.11 to 0.33).

### 3.2 The nPLN and LNR exhibit a compelling prognostic relationship at particular threshold values (nPLN at 1 and 2; LNR at 0.17 and 0.38)

**FIGURE 2:**
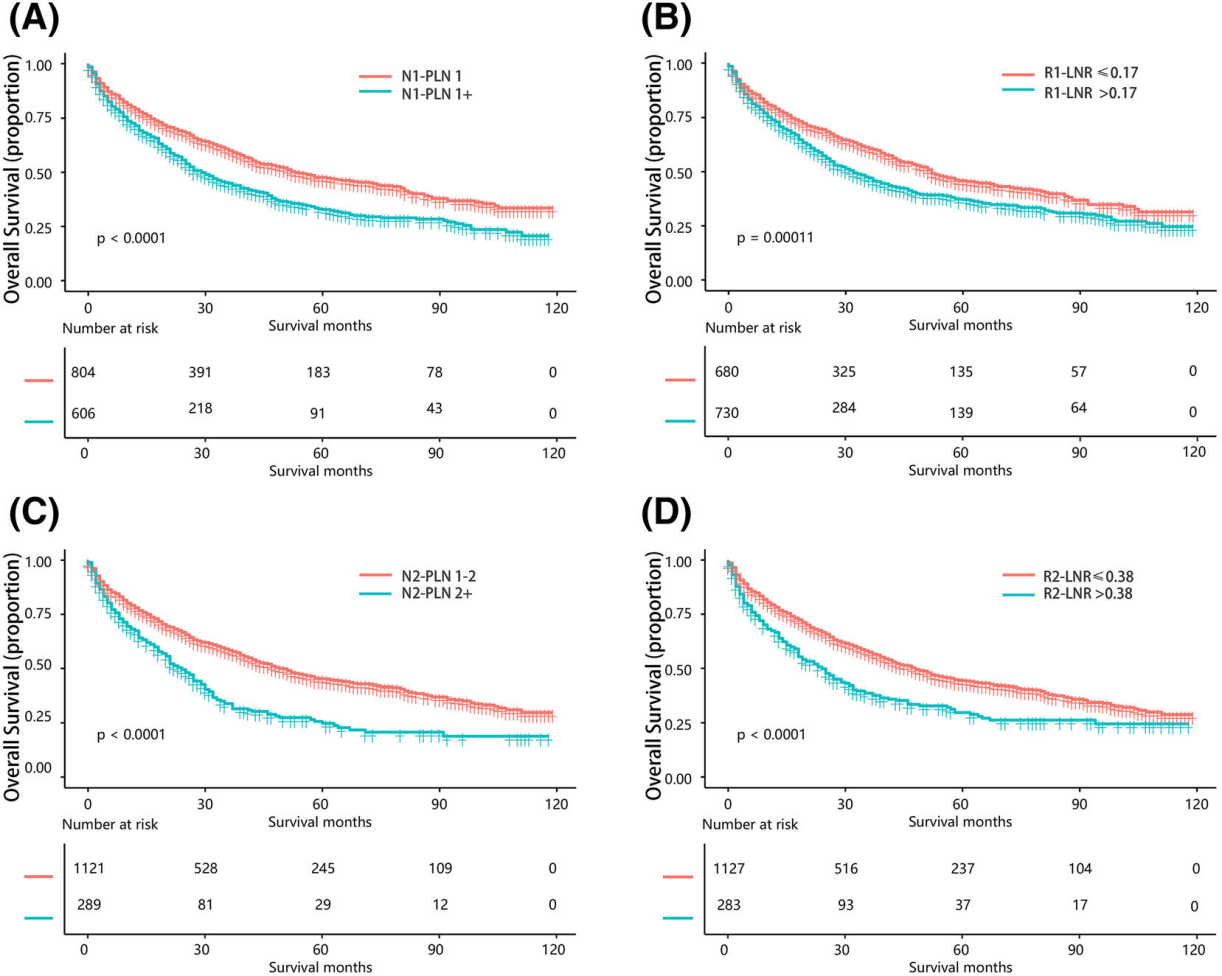
(A) KM curves according to the nPLN subgroup in patients with cutoff 1. (B) KM curves according to the LNR subgroup in patients with cutoff 0.17. (C) KM curves according to the nPLN subgroup in patients with cutoff 2. (D) KM curves according to the LNR subgroup in patients with cutoff 0.38. KM: Kaplan–Meier, nPLN: number of positive lymph nodes, LNR: positive lymph node rate.

We evaluated the prognostic significance of nPLN and LNR in relation to the current N staging system by analyzing survival curves. Our results revealed significant differences in overall survival between subgroups (Figure 2), with 5‐year survival rates of 47.32% and 33.14% for N1‐PLN 1 and N1‐PLN 1+, 45.03% and 25.44% for N2‐PLN 1–2 and N2‐PLN 2+, 49.63% and 37.52% for R1‐LNR ≤0.17 and R1‐LNR >0.17, and 44.1% and 31.4% for R2‐LNR of 0.38 and R2‐LNR >0.38, respectively. The statistical significance of these differences was confirmed by *P* values <0.001 for all comparisons.

### 3.3 The impact of nPLN or LNR categories on patient survival was evaluated in this study

**FIGURE 3:**
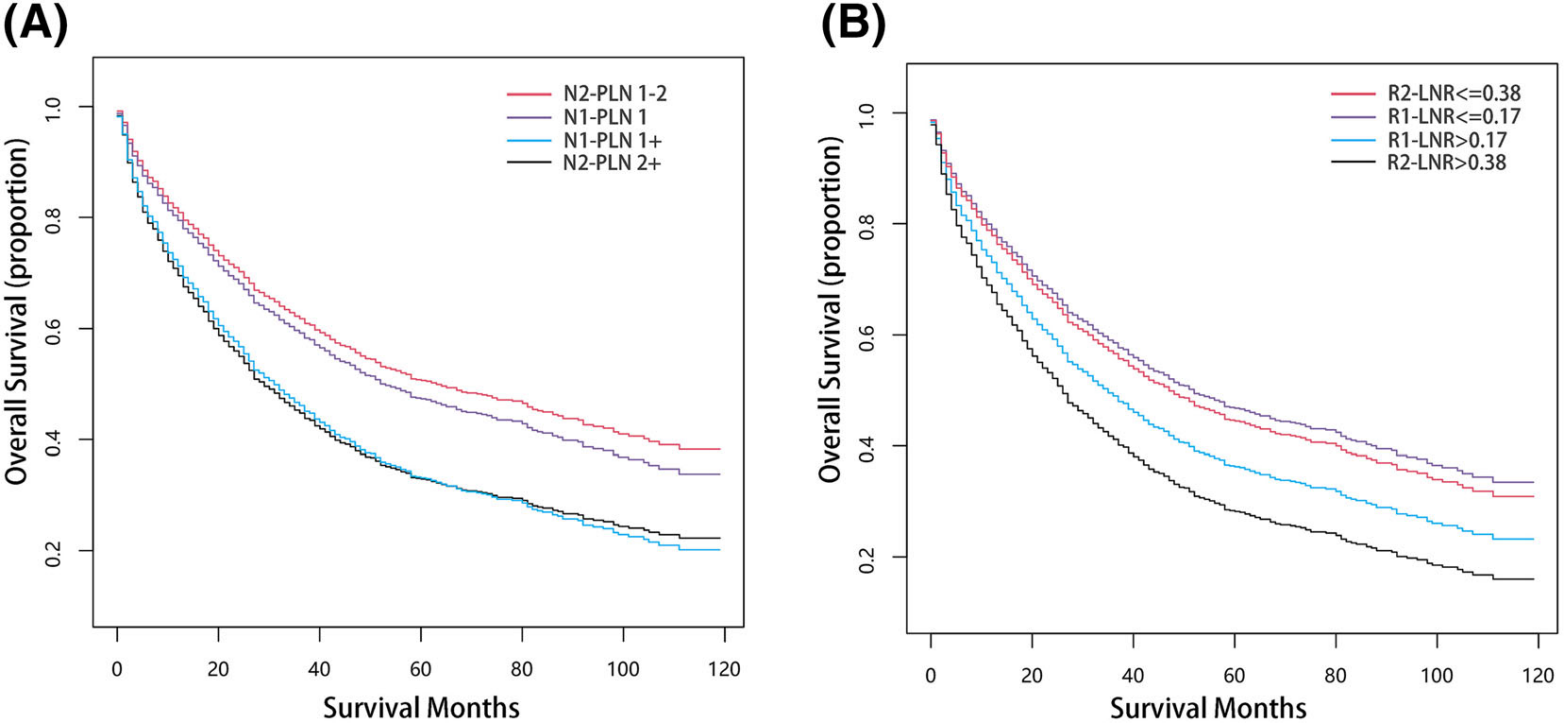
(A) KM curves according to the nPLN subgroup in patients with cutoff 1 and 2. (B) KM curves according to the LNR subgroup in patients with cutoff 0.17 and 0.38. KM: Kaplan–Meier, nPLN: number of positive lymph nodes, LNR: positive lymph node rate.

We classified patients into four distinct subgroups based on their nPLN or LNR categories and examined their survival outcomes using survival curves (Figure 3). Two sets of survival curves based on nPLN and LNR are plotted in Figure 2 based on individual structural breakpoints, and in Figure 3, we have merged these two sets of survival curves for observation respectively. Our analysis revealed that there were significant differences in OS (*P* < 0.001) between each pair of nPLN categories, with the exception of N1‐PLN 1+ and N2‐PLN 2+. Similarly, statistically significant differences in OS were observed between each pair of LNR categories, with significant differences noted between adjacent LNRs (*P* < 0.001).

### 3.4 Investigating the influence of novel nPLN and LNR classification

**FIGURE 4:**
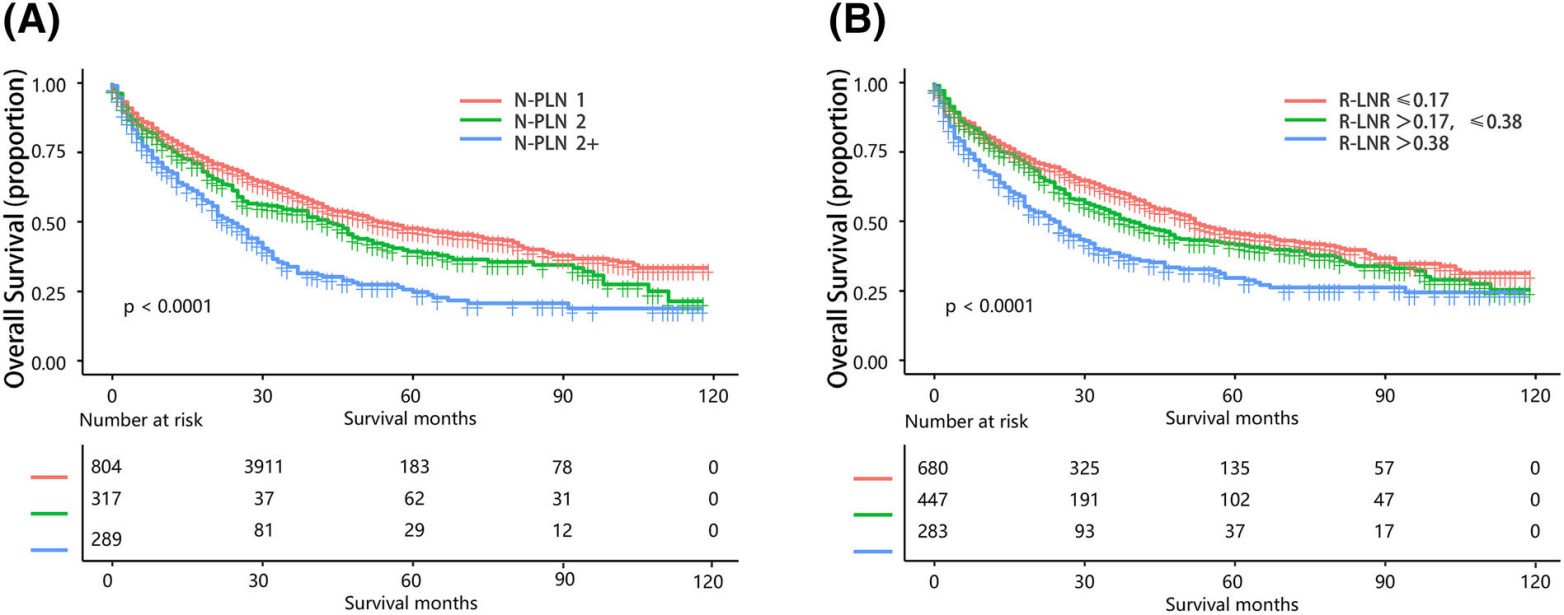
(A) KM curves according to the new nPLN subgroup in all patients. (B) KM curves according to the new LNR subgroup in all patients. KM: Kaplan–Meier, nPLN: number of positive lymph nodes, LNR: positive lymph node rate.

Figure 3 depicted survival curves that exhibited an overlap between various nPLN and LNR categories, including N1‐PLN 1, N1‐PLN 1+, N2‐PLN 1–2, N2‐PLN 2+, R1‐LNR ≤0.17, R1‐LNR >0.17, R2‐LNR ≤0.38, and R2‐LNR >0.38. To address this issue, we proposed new classification criteria based on N‐PLN and R‐LNR to group together survival curves that displayed similar patterns. The new nPLN categories included N‐PLN 1, N‐PLN 2, and N‐PLN 2+, while the new LNR categories comprised R‐LNR ≤0.17, R‐LNR >0.17, ≤0.38, and R‐LNR >0.38. Figure 4 showed positively distributed survival curves for each new nPLN and LNR category, revealing a gradual decrease in the 5‐year overall survival (OS) rates with increasing nPLN and LNR values (47.22%, 39.58%, and 25.42% for N‐PLN 1, N‐PLN 2, and N‐PLN 2+, respectively; *P* < 0.001 for all comparisons).

**FIGURE 5:**
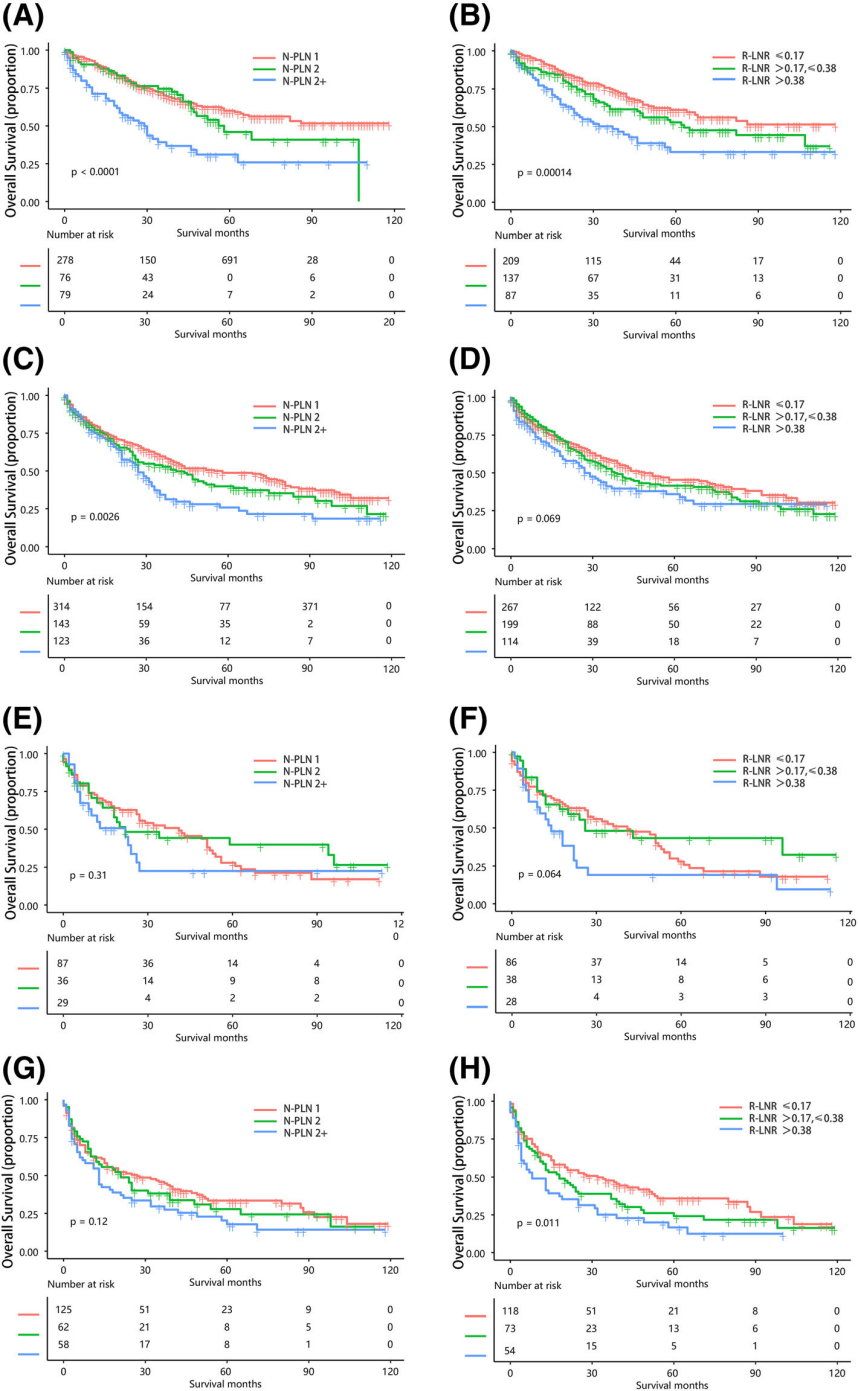
(A) KM curves according to the new nPLN classification of patients with tumor size ≤3 cm. (B) KM curves according to the new LNR classification of patients with tumor size ≤3 cm. (C) KM curves according to the new nPLN classification of patients with tumor size >3 cm, ≤5 cm. (D) KM curves according to the new LNR classification of patients with tumor size >3 cm, ≤5 cm. (E) KM curves according to the new nPLN classification of patients with tumor size >5 cm, ≤7 cm. (F) KM curves according to the new LNR classification of patients with tumor size >5 cm, ≤7 cm. (G) KM curves according to the new nPLN classification of patients with tumor size >7 cm. (H) KM curves according to the new LNR classification of patients with tumor size >7 cm. KM: Kaplan–Meier, nPLN: number of positive lymph nodes, LNR: positive lymph node rate.

We then evaluated the impact of the novel nPLN and LNR categories on survival across a range of tumor sizes (3, 3–5, 5–7, and >7 cm). Survival curves were significantly different between each pair of nPLN categories for all tumor sizes, except for N‐PLN 1 and N‐PLN 2 in tumors 5–7 and >7 cm (Figure 5). However, for LNR categories, significant differences in OS were observed only for tumors <3 and ≥7 cm, while no significant difference in OS was noted between LNR categories for tumors 3–7 cm. Moreover, in tumors 5–7 cm, survival curves for R‐LNR ≤0.17 and R‐LNR >0.17, ≤0.38 were found to overlap (Figure 5).

In our univariate analysis (Table 2), variables significantly associated with patient OS in the univariate analysis will be included as covariates in the multivariable analysis, with adjustments made for their confounding effects. All three nodal block classifications were identified as significant independent prognostic markers based on multifactorial Cox regression analysis. Notably, both nPLN and LNR classifications displayed high Harrell consistency index (C‐index), Akaike information criterion (AIC), and Bayesian information criterion (BIC) (Table 3). Furthermore, compared with the nPLN classification, the LNR classification provided superior prognostic stratification, as indicated by the hazard ratios (HRs) of 1.11 for N‐PLN 2 (*P* = 0.2743) and 1.24 for R‐LNR >0.17, ≤0.38 (*P* = 0.0140), as well as the HRs of 1.71 for N‐PLN 2+ (*P* = <0.0001) and 1.80 for R‐LNR >0.38 (*P* < 0.0001), respectively (Table 3). Model I adjusted for: None. Model II model adjusted for: Age; Sex; Race (except for the variables itself). Model III adjusted for: Age; Sex; Race; Primary site; Grade; Histology; Operation type; Tumor size; Postoperative radiation; ELN continuous; Positive lymph notes continuous (except for the variables itself). The results presented by the three models did not show significant differences, demonstrating that the results of N staging, nPLN classification, and LNR classification were all less affected by the aforementioned covariates.

**TABLE 2:** Univariate analyses for overall survival in all patients.

Variable	Univariate
	HR (95% CI) *P*
Age	
<65 years	Reference
≥65 years	1.9 (1.6, 2.2) < 0.001
Sex	
Male	Reference
Female	0.7 (0.6, 0.8) < 0.001
Race	
White	Reference
Black	0.8 (0.6, 1.1) 0.129
Other	0.8 (0.6, 1.0) 0.067
Primary_site	
Upper lobe	Reference
Middle lobe	0.7 (0.5, 1.0) 0.057
Lower lobe	0.9 (0.8, 1.1) 0.206
Grade	
I	Reference
II	4.5 (3.2, 6.4) < 0.001
III–IV	5.7 (4.0, 8.1) < 0.001
Histology	
SCC	Reference
ADC	0.8 (0.7, 1.0) 0.019
Other	0.5 (0.4, 0.6) < 0.001
Operation type	
Lobectomy	Reference
Pneumonectomy	1.7 (1.4, 2.2) < 0.001
Tumor size	
≤3 cm	Reference
>3 cm, ≤5 cm	1.5 (1.2, 1.8) < 0.001
>5, ≤7 cm	2.0 (1.5, 2.5) < 0.001
>7 cm	2.3 (1.9, 2.9) < 0.001
Postoperative radiation	
Yes	Reference
No	1.1 (0.8, 1.5) 0.659
Current N classification	
N1	Reference
N2	1.0 (0.8, 1.3) 0.798
Positive lymph notes	
1	Reference
2	1.2 (1.0, 1.5) 0.023
2+	1.8 (1.5, 2.2) < 0.001
LNR categorical	
≤0.17	Reference
>0.17, ≤0.38	1.2 (1.0, 1.4) 0.078
>0.38	1.7 (1.4, 2.0) < 0.001
C‐index	
AIC	
BIC	

Abbreviations: ADC, adenocarcinoma; CI: confidence interval.; ELN, examined lymph node; HR: hazard ratio; LNR, lymph nodes ratio; SCC, squamous cell carcinoma.

**TABLE 3:** Multivariate analyses and model evaluation for overall survival in all patients.

Variable	Multivariate		
	Model I	Model II	Model III
	HR (95% CI) *P*	HR (95% CI) *P*	HR (95% CI) *P*
Current N classification			
N1	Reference	Reference	Reference
N2	1.04 (0.79, 1.35) 0.7984	1.14 (0.87, 1.49) 0.3426	1.03 (0.78, 1.36) 0.8320
Positive lymph notes			
1	Reference	Reference	Reference
2	1.23 (1.03, 1.48) 0.0229	1.17 (0.98, 1.40) 0.0865	1.11 (0.92, 1.33) 0.2743
2+	1.81 (1.52, 2.17) <0.0001	1.77 (1.48, 2.12) <0.0001	1.71 (1.43, 2.06) <0.0001
LNR categorical			
≤0.17	Reference	Reference	Reference
>0.17, ≤0.38	1.16 (0.98, 1.38) 0.0779	1.21 (1.02, 1.43) 0.0286	1.24 (1.04, 1.47) 0.0140
>0.38	1.65 (1.37, 1.99) <0.0001	1.71 (1.42, 2.06) <0.0001	1.80 (1.49, 2.18) <0.0001
**Model evaluation**	**Current N classification**	**PLN categorical**	**LNR categorical**
C‐index	0.505	0.5628	0.5578
AIC	1712	1691	1687
BIC	1785	1769	1766

*Note*: Model I adjusted for: None. Model II model adjusted for: Age; Sex; Race (except for the variables itself). Model III adjusted for: Age; Sex; Race; Primary site; Grade; Histology; Operation type; Tumor size; Postoperative radiation; ELN continuous; Positive lymph notes continuous (except for the variables itself).

Abbreviations: AIC: Akaike information criterion; BIC: Bayesian information criterion; CI: confidence interval; HR: hazard ratio; LNR, lymph nodes ratio.

## 4 DISCUSSION

The findings of this study reveal that patients with a higher nPLN and higher LNR exhibit a higher risk of death and recurrence.^23^, ^24^, ^25^, ^26^, ^27^ Nwogu et al. conducted a study on all NSCLC patients who underwent curative resection and had at least one LN examined in the SEER database from 1988 to 2007.^9^ They discovered that an increase in the number of removed LNs or the proportion of metastatic LNs to total LNs improved the prognosis of NSCLC. In this study, we aimed to examine the potential for improved survival prediction by stratifying patients into subgroups based on nPLN and LNR. However, we found that LNR was a more significant prognostic factor than N category, as an increase in LNR was linked with a decrease in OS in N1 and N2 patients. Furthermore, we discovered that adding nPLN or LNR categories to the N category could result in a more precise survival prediction, as relying solely on location‐based N categorization has limited predictive and discriminating power.

To further categorize N1 or N2 diseases into subgroups and improve the accuracy of survival prediction, we propose that the nPLN and LNR categories be added to the N category. These additional prognostic data go beyond location‐based N classification. However, we acknowledge that there is limited research on the combined effect of N category and nPLN or LNR on prognosis, and it remains unclear whether nPLN or LNR categories are superior prognostic indicators. In this study, we used a large, nationally representative cohort of N1 or N2 resected patients to thoroughly examine the effects of current N staging, nPLN staging, and LNR staging on survival and fill the knowledge gap. We compared various staging approaches to identify the method that most accurately predicts survival outcomes for N1 and N2 patients.

In the total population, the survival curves for both the nPLN subgroup and the LNR subgroup showed significant differences. However, in the subgroup analyses grouped by tumor size, survival curves were significantly different between each pair of nPLN categories for all tumor sizes, except for N‐PLN 1 and N‐PLN 2 in tumors 5–7 cm and >7 cm (Figure 5). It may be that the prognosis of large tumors is already poor regardless of lymph node status. For LNR categories, significant differences in OS were observed only for tumors <3 cm and ≥7 cm, while no significant difference in OS was noted between LNR categories for tumors 3–7 cm, this may be due to the small size of the population. Therefore, this subgroup analysis did not adequately demonstrate the impact of nPLN and LNR on survival in various tumor size categories. The study by Li et al.^8^ also found that for large tumors (>7 cm), the difference in survival curves between nPLN and LNR classification was not significant. This may also be due to the poor prognosis of larger tumors.

Our study highlights the potential for improved survival prediction by incorporating nPLN and LNR categories into the N category for N1 and N2 diseases. The results indicate that LNR is a more significant prognostic factor than the N category, and further investigation is required to determine the combined effect of N category and nPLN or LNR on prognosis. By employing a substantial cohort of N1 or N2 resected patients, we were able to comprehensively compare different staging methods and provide insights into the most accurate method for survival prediction in these patient populations.

Our study endeavors to evaluate the prognostic significance of LN‐related classifications in cancer patients. By employing optimal cutoff values for nPLN and LNR, we partitioned our patient cohort into N‐PLN and R‐LNR categories. Our meticulous analysis divulged noteworthy variations in overall survival OS curves between nearly all pairs of categories, thereby underscoring the potential utility of these classifications for prognostic stratification. Nonetheless, certain exceptions existed where the OS curves failed to evince significant differences, prompting us to suggest novel nPLN and LNR categories. Subsequently, we utilized these novel categories to recategorize our patient cohort into three subgroups, which demonstrated substantial discrepancies and proportionate connections between each pair of the nPLN and LNR categories. Our results intimate that LNR classification may outperform nPLN classification in terms of prognosis. Specifically, we noted that OS plummeted significantly from N‐PLN 1 to N‐PLN 2+ across tumor size categories of 5 cm; however, dissimilarities between each pair of R‐LNR categories manifested significance solely in tumors measuring ≤3 and >7 cm.

Our study aimed to evaluate the prognostic value of LN‐related classifications for patients with cancer using a multivariate analysis. We found that all three classifications, N, nPLN, and LNR, were independent prognostic factors. However, we discovered that the nPLN and LNR classifications outperformed the current N classification based on their respective values of C‐index, AIC, and BIC. Interestingly, LNR showed a superior prognostic effect compared with nPLN.

Our study also revealed a limitation of the current AJCC TNM staging system for lung cancer, which does not consider the absolute number of involved nodes and LNs or LN stations examined. To address this limitation, we included a threshold of 16 LNs removed in our study based on previous research.^30^ We believe that this criterion is an essential consideration for future studies and the development of a more accurate staging system.

This study has several limitations that should be considered when interpreting the results. First, the retrospective nature of the analysis may have introduced bias into the study and may have compromised the validity of the analysis. Additionally, the potential for selection bias cannot be completely ruled out. While the individuals responsible for inputting the data were blind to the assumptions of this study, there may have been other factors that influenced the selection of patients for inclusion in the analysis. Second, the lack of standardization in LN assessment may have limited the ability to standardize LN assessment for all patients undergoing resection. Surgeons' bias regarding the role of LN sampling versus LN dissection may have introduced variation in the LN assessment process. Third, there was no standardized pathological criteria for LN counting and assessment, which may have resulted in differences in LN counts between different institutional pathologists. Although this potential limitation was addressed by having the same pathologist evaluate LNs removed at the time of resection, it cannot be completely ruled out. Another limitation of this study was the frequency of patient recurrence assessment. Patient recurrence assessment was performed every 3 to 6 months, and the specific time of recurrence was determined based on the time between assessments, which may have resulted in inaccuracies in determining the exact time of recurrence. Finally, the SEER database did not include information on certain potential confounding factors such as comorbid conditions, performance status scores, tumor volume, use of systemic therapy, or subsequent local therapy, which may have limited the generalizability of these findings. Further studies with larger sample sizes and more comprehensive data are needed to address these limitations and to confirm the prognostic value of the LN‐related classifications.

Furthermore, we contend that LNR classification is a more accurate indicator of the degree of LN involvement. Unlike nPLN, LNR accounts for the effect of ELN, which also has a prognostic impact. Our analysis showed that LNR demonstrated better prognostic performance compared with nPLN. Therefore, LNR may be a more desirable extra N descriptor for the upcoming staging system version.

## 5 CONCLUSIONS

To summarize, our study highlights the significant prognostic value of nPLN and LNR in predicting survival outcomes for LN‐positive NSCLC patients who undergo surgery. Our results demonstrate that these classifications may offer improved prognostic performance compared with the current N classification. Nonetheless, more studies are needed to validate our findings and assess the feasibility of incorporating these classifications into the next TNM staging system.

## AUTHOR CONTRIBUTIONS

*Conception and design*: Qiang Guo and Yiping Wei. *Administrative support*: Qiang Guo and Sheng Hu. *Provision of study materials or patients*: Qiang Guo and Lang Su. *Collection and assembly of data*: Qiang Guo. *Data analysis and interpretation*: Qiang Guo, Silin Wang, and Wenxiong Zhang. *Manuscript writing*: Qiang Guo. *Final approval of manuscript*: Qiang Guo, Sheng Hu, Silin Wang, Yiping Wei, Jianjun Xu, Wenxiong Zhang.

## CONFLICT OF INTEREST STATEMENT

The authors declare that the research was conducted in the absence of any commercial or financial relationships that could be construed as a potential conflict of interest.

## ETHICS STATEMENT

The study protocol was approved by the ethics committee of the Second Hospital of Nanchang University in China, ensuring that the study was conducted in accordance with ethical principles and regulations.

## Supporting information


**Figure S1:** The best cut‐off values for the nPLN and LNR by using X‐tile. a) The cut‐point for the entire queue of nPLN. b) The histogram of the entire cohort of nPLN. c) The relative risk of the entire queue of nPLN. d) The cut‐point for the entire queue of LNR. e) The histogram of the entire cohort of LNR. f) The relative risk of the entire queue of LNR. nPLN: Number of positive lymph nodes, LNR: Positive lymph node rate.

## Data Availability

The datasets analyzed for this study can be found in the SEER database (https://seer.cancer.gov/).
