# Supplementary figures and images for: Comparative analysis of methodologies for predicting overall survival in patients with non‐small cell lung cancer based on the number and rate of resected positive lymph nodes: A study based on the SEER database for 2010 through 2019
Source: Clin Respir J. 2023 Sep 18;17(11):1145–57. doi: 10.1111/crj.13699 (PMC10632082; doi:10.1111/crj.13699)

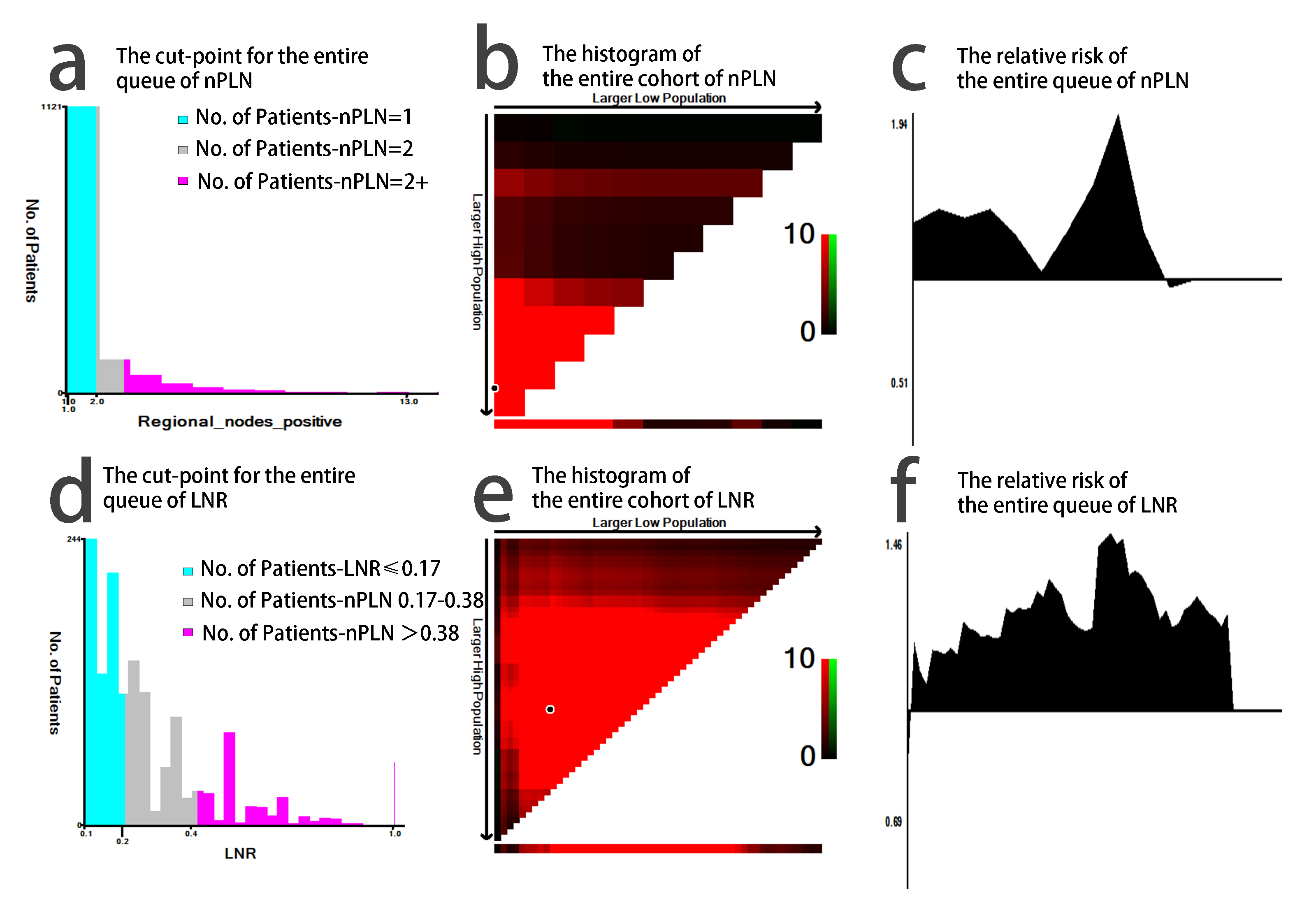

Supplement: Supplementary file 1 — Figure S1: The best cut‐off values for the nPLN and LNR by using X‐tile. a) The cut‐point for the entire queue of nPLN. b) The histogram of the entire cohort of nPLN. c) The relative risk of the entire queue of nPLN. d) The cut‐point for the entire queue of LNR. e) The histogram of the entire cohort of LNR. f) The relative risk of the entire queue of LNR. nPLN: Number of positive lymph nodes, LNR: Positive lymph node rate. [file CRJ-17-1145-s001.tif]
